# Biointerfaces with ultrathin patterns for directional control of cell migration

**DOI:** 10.1186/s12951-024-02418-3

**Published:** 2024-04-08

**Authors:** Yijun Cheng, Stella W. Pang

**Affiliations:** 1 Department of Electrical Engineering, City University of Hong Kong, Kowloon, Hong Kong China; 2 Centre for Biosystems, Neuroscience, and Nanotechnology, City University of Hong Kong, Kowloon, Hong Kong China

**Keywords:** Unidirectional migration, Patterned titanium oxide, Microelectrode, Cell monitoring, MC3T3-E1 cell

## Abstract

In the context of wound healing and tissue regeneration, precise control of cell migration direction is deemed crucial. To address this challenge, polydimethylsiloxane (PDMS) platforms with patterned 10 nm thick TiO_x_ in arrowhead shape were designed and fabricated. Remarkably, without tall sidewall constraints, MC3T3-E1 cells seeded on these platforms were constrained to migrate along the tips of the arrowheads, as the cells were guided by the asymmetrical arrowhead tips which provided large contact areas. To the best of our knowledge, this is the first study demonstrating the use of thin TiO_x_ arrowhead pattern in combination with a cell-repellent PDMS surface to provide guided cell migration unidirectionally without tall sidewall constraints. Additionally, high-resolution fluorescence imaging revealed that the asymmetrical distribution of focal adhesions, triggered by the patterned TiO_x_ arrowheads with arm lengths of 10, 20, and 35 μm, promoted cell adhesion and protrusion along the arrowhead tip direction, resulting in unidirectional cell migration. These findings have important implications for the design of biointerfaces with ultrathin patterns to precisely control cell migration. Furthermore, microelectrodes were integrated with the patterned TiO_x_ arrowheads to enable dynamic monitoring of cell migration using impedance measurement. This microfluidic device integrated with thin layer of guiding pattern and microelectrodes allows simultaneous control of directional cell migration and characterization of the cell movement of individual MC3T3-E1 cells, offering great potential for the development of biosensors for single-cell monitoring.

**Supplementary Information:**

The online version contains supplementary material available at 10.1186/s12951-024-02418-3.

## Introduction

Tissue engineering is a rapidly advancing field that focuses on restoring damaged tissues using a combination of cells, scaffolds, and biochemical or mechanical signals [1, 2]. To achieve successful tissue regeneration, precise control of cell migration direction is essential. For instance, in the case of treating severe bone defects, it is crucial to induce cells to migrate in a well-ordered manner from the proximal to distal region, promoting directional bone growth [3, 4]. Therefore, optimizing the surface conditions of bone implants to promote osteoblast cell positioning, alignment, and migration direction is essential for repairing and replacement of damaged bone tissue.

Various biochemical and biophysical cues, such as chemical concentration, stiffness, surface energy, and topography have been developed to control cell migration [2, 5, 6]. These cues can lead to changes in cell shape, cytoplasmic or nuclear properties, actomyosin machinery, and migration strategy [7–9]. For example, in vitro concentration gradients of surface-bound proteins, small ligands, and growth factors have been used to study their influence on cell migration. One study found that smooth muscle cells aligned themselves toward the direction of a basic fibroblast growth factor (bFGF) concentration gradient and moved toward the area with higher concentration of bFGF [10]. However, chemical gradients can be degraded over time and are based on cell-specific chemoattractants that are often unknown for most cell types. Physical stimuli, such as stiffness gradients on a compliant substrate, can also guide directional cell migration [11]. Cell polarization is tightly regulated by various cues that operate through controlled recruitment of signaling complexes. These complexes play a crucial role in modulating the actin cytoskeleton and facilitating the extension of membrane protrusions at the leading edge. During directional migration, the establishment and maintenance of cell polarization are mediated by a combination of positive and negative feedback mechanisms. These mechanisms involve integrins, phosphoinositides, cytoplasmic adaptor proteins, and Rho family GTPases. However, chemical and physical cues often have limitations in their effectiveness and range.

Surface energy can play a significant role in facilitating new bone formation by influencing the response of osteoblasts to materials [12, 13]. Studies have indicated that titanium surface with high surface energy can enhance osteoblast migration and differentiation [14]. In addition, a study investigated the interaction between mesenchymal stem cells (MSCs) and soft biomaterial substrates [15]. The findings of their study demonstrate that the assembly of surface-driven ligands can influence the MSCs’ perception of the mechanical properties of the substrate. The use of substrates with varying surface energies resulted in the formation of different ligand topologies, which in turn influenced the self-assembly of surface proteins and led to the activation of distinct transmembrane cell receptors and adhesion signals. Although these differences did not directly affect the activity of Rho-associated kinases, they did regulate cell diffusion and subsequent differentiation. Additionally, surface energy can regulate the migration speed and morphology of MC3T3-E1 cells [13]. Various surface topographies and coatings were employed to investigate their impact on cell migration behaviors. These surfaces included flat polydimethylsiloxane (PDMS), nanopillar, silicon oxide (SiO_x_), and titanium oxide (TiO_x_). The flat PDMS or nanopillar surfaces exhibited hydrophobic properties, while the SiO_x_ and TiO_x_ coatings rendered the surfaces hydrophilic. Notably, cells on hydrophobic surfaces with lower surface energy displayed higher migration speeds and greater elongation than those on hydrophilic surfaces with higher surface energy. Even after FN coating, the migration speed of cells on nanopillars remained significantly distinct from that of cells on other surfaces. These findings underscore the influence of FN adsorption dependence on surfaces with different surface energies on migration behaviors. These studies emphasize the significance of considering the surface energy as a crucial design factor.

Surface topography also plays a crucial role in cell-material interactions by influencing cell orientation and migration [16–20]. Gratings with specific width and depth have been utilized to guide bidirectional cell migration along grooves and ridges [21]. Osteoblasts cultured on microgratings with a height of 7 μm and varying widths (10, 15, and 30 μm) exhibited significantly enhanced polarization morphology compared to those on flat surfaces. However, symmetrical gratings resulted in bidirectional cell movement, which is less beneficial for tissue repair where unidirectional cell migration holds greater significance. Furthermore, bent gratings were designed as unidirectional switching gates to control cell migration direction, which involved two key parameters: the sharpness of the bend and its position relative to the end [22]. The bending angles were 45°, 90°, and 135°. When the bending angles were set at 90° and 135°, cells had two migration options: they could either move along the grating through the bend or reverse their direction at the bend. In contrast, when the bending angle was 45°, cells exhibited the ability to form large cytoplasmic protrusions around the curved tips and migrate in the direction of the arrowhead tips, thus creating a third migration path along the tip direction. Despite the effectiveness of bent microgratings in promoting cell unidirectional movement, a notable limitation arises in the challenge of collecting cells on the patterned surface. Another limitation lies in the incapacity to guide cells for prolonged distances in a single direction. The current configuration in this study provides extended unidirectional guidance for cell migration over substantial lengths to direct cellular movement toward specific locations. Gratings and arrowheads in channels enclosed by 12 μm tall channel walls have been used to guide cell migration to climb vertically over steps [23]. In this study, no physical walls were built to constrain the cells. Instead, arrowhead patterns defined by a thin layer of TiO_x_ were used to provide the unidirectional cell migration control. Cells could recognize surface topography and respond accordingly, potentially through reshaping of the nucleus and cytoskeleton [24, 25]. We plan to study how biointerfaces with thin layer of guiding patterns could affect cell migration direction.

TiO_x_ was selected in this study due to its exceptional biocompatibility and long-term stability. It does not compromise cellular or tissue function upon contact with bodily fluids, and it is associated with minimal adverse reactions, such as inflammation, cancer, or rejection. Consequently, it is commonly employed as coatings for bone grafts to stimulate new bone formation [26]. Additionally, contact angle of TiO_x_ exhibits 74° which helps to keep cells on the TiO_x_ patterns and away from the PDMS surface because cell adhesion was the highest for contact angles between 60° and 80° [14, 27, 28]. Moreover, TiO_x_ can be conveniently deposited not only on 2D surfaces but also on complex 3D structures using nanofabrication technology. This versatility opens up possibilities for selectively coating 3D platforms, thereby promoting cell migration and tissue repair in 3D.

Microfluidic devices have proven invaluable in the study of high-throughput cell migration. A novel microfluidic device featuring five continuously bifurcated channels was developed to investigate the directionality of cancer cells during migration on a self-generating epidermal growth factor gradient [29]. This device enables the analysis of new relationships between different chemical components and cell orientation during migration. Additionally, a specially designed microfluidic chip facilitates multifunctional drug screening against the mass migration of cancer cells within a confined environment [30]. These microfluidic devices offer an effective method for high-throughput screening of drugs. Furthermore, bioactive ceramic microfluidic chips were employed to examine cell migration across various ceramic microbridges [31]. These chips enable the visual and dynamic observation of migration differences among cells in distinct ceramic microbridges. This model provides simulation of in vivo environments and offers greater control over input-output conditions compared to other cell migration detection methods. Moreover, a high-throughput microfluidic cell migration platform was developed with integrated robotic liquid handling and computer vision to facilitate rapid quantification of individual cell movements [32]. This platform had successfully resolved correlations between cell migration and various growth inhibition factors for drug screening. However, there are some limitations in these studies. First, external fluid flow is required, which could influence cell migration. Second, the analysis of cell migration behavior relies on optical microscopy, which poses challenges for equipment miniaturization and automated analysis.

On the other hand, impedance spectroscopy is a non-invasive technique to characterize the electrical properties of biological tissue and cells [33]. It provides valuable information on membrane resistance, capacitance, and cytoplasmic conductivity at frequencies below 10 MHz [34]. While bulk suspensions have been used to study cell behaviors, they only provide population averages and cannot capture individual cell heterogeneity [35]. As a result, techniques with single-cell resolution have been investigated [36, 37]. By combining impedance spectroscopy with a microfluidic chip, label-free impedance characterization of single cells can be performed.

In this study, the ability of patterned TiO_x_ to guide MC3T3-E1 cells in a single direction without tall sidewall constraints was investigated. PDMS platforms were fabricated using a liftoff technology with water-soluble polyvinyl alcohol (PVA) as a sacrifice layer, which provided smooth edges and avoided the use of corrosive reagents or organic solvents that could damage the PDMS substrate [38–40]. Cells were seeded on PDMS platforms with a 10 nm thick layer of patterned TiO_x_, allowing them to migrate along the arrowhead tips. Unlike previous methods that relied on tall sidewall constraints to control cell migration direction, the thin TiO_x_ arrowhead pattern in combination with a PDMS surface were utilized. This novel technique demonstrated the first successful control of unidirectional cell migration without tall sidewall constraints. These findings have significant implications for the design of biointerfaces with ultrathin patterns to facilitate tissue repair and regeneration. To enable dynamic monitoring of cell migration, gold (Au)/Chromium (Cr) microelectrodes were integrated with the patterned TiO_x_ arrowheads. This allowed for impedance measurement to characterize the electrical properties of cells and tissues. An integrated microfluidic device with microelectrodes was developed to characterize the movement of individual MC3T3-E1 cell. These innovations represent important advancements in the field of simultaneously controlling cell migration and studying cell movement, and have the potential to significantly improve our understanding of cellular behavior in tissue engineering applications.

## Experiment and methods

### Fabrication technology for patterning TiO_x_ on PDMS substrate

**Fig. 1:**
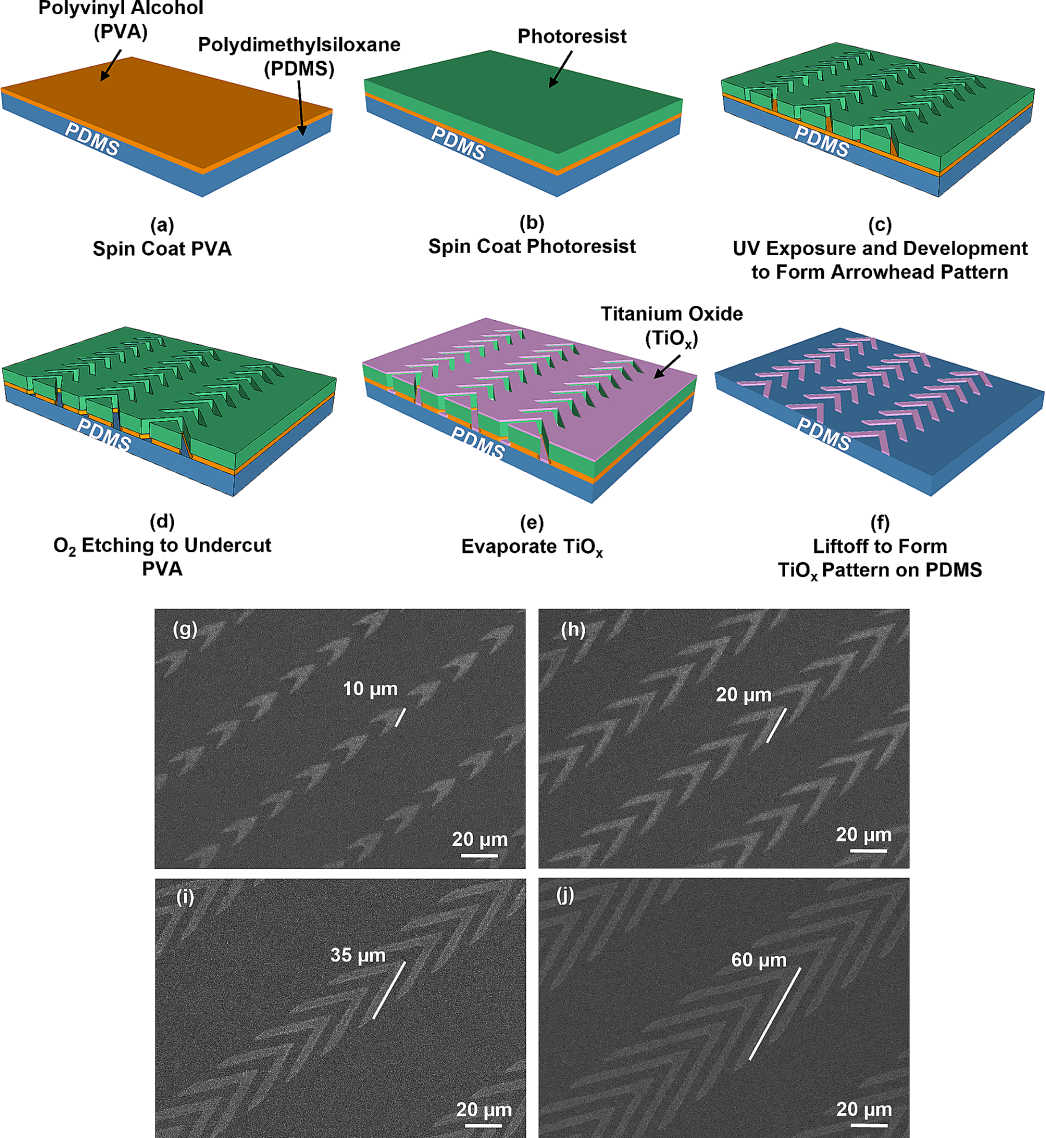
Fabrication technology and micrographs of patterned titanium oxide (TiO_x_) on polydimethylsiloxane (PDMS) substrate. (**a**) Spin-coating of polyvinyl alcohol (PVA) film onto PDMS substrate, followed by drying on hotplate. (**b**) Spin-coating of photoresist onto PVA film. (**c**) Transfer of patterns onto PVA surface using UV lithography. (**d**) Reactive ion etching (RIE) PVA film to form undercut profile. (**e**) Deposition of thin layer of TiO_x_ on PDMS using electron beam evaporation. (**f**) Formation of patterned TiO_x_ on PDMS substrate by liftoff. Micrographs of patterned TiO_x_ in arrowhead shape are shown, with arrowhead arms of lengths of (**g**) 10, (**h**) 20, (**i**) 35, and (**j**) 60 μm. The arrowhead arms were 5 μm wide with 5 μm separation, and arrowhead tip angle was 45°

Figure 1(a-f) illustrate the fabrication technology of TiO_x_ patterned on PDMS substrate. Glass substrate was thoroughly cleaned with acetone, isopropanol, and deionized (DI) water for 10 minutes (min), and then baked at 110 ℃ for 10 min. To enhance the hydrophilicity of the glass substrate, it underwent an O_2_ plasma treatment for 30 s using 100 sccm O_2_, 0.15 mbar pressure, and 75 W radio frequency (RF) power. Next, PDMS (base: crosslinker weight ratio = 10:1, Sylgard 184, Dow Corning, USA) was spin-coated onto the glass substrate at 5000 rotation per min (rpm) for 60 seconds (s), degassed and baked at 120 ℃ for 6 hours (h). The glass substrate coated with PDMS was then subjected to an O_2_ plasma treatment for 30 s using 100 sccm O_2_, 0.15 mbar, and 75 W RF power. A 4% (weight by volume) PVA (Invitrogen, USA) was spin-coated on the PDMS substrate at 3000 rpm for 60 s and then baked on a hotplate at 110 ℃ for 2 min to form a 200 nm thick film, as depicted in Fig. 1(a). After cooling for 5 min, a positive photoresist was spin-coated on the PVA film at 3000 rpm for 60 s, as shown in Fig. 1(b). The sample was then soft-baked at 105 ℃ for 15 min, exposed to ultraviolet (UV) light for 12 s through a photomask, developed in AZ 300 MIF developer for 24 s, and hard-baked at 120 ℃ for 10 min, as shown in Fig. 1(c), to form the arrowhead pattern.

Reactive ion etching (RIE) (790 RIE system, Plasma-Thermal, USA) was used to etch the PVA with a condition of 20 sccm O_2_, 40 mTorr, and 100 W RF power for 5 min. The etch rates for PVA and photoresist were 165 and 80 nm/min, respectively, resulting in an undercut profile after O_2_ etching, as shown in Fig. 1(d). 10 nm thick Ti was deposited on the platform using electron beam evaporation (ATS 500, HHV, UK) with deposition rate of 6.0 nm/min, as shown in Fig. 1(e). The platform was then ultrasonicated in DI water for 20 min to dissolve the PVA sacrificial layer. After blow-drying the platform with nitrogen, the patterned TiO_x_ was formed after baking at 110 ℃ for 10 min and the platform was stabilized after two days at 25 ℃, as shown in Fig. 1(f). Supplementary Fig. S1 shows changes of hydrophobicity observed on different surfaces over time. For the TiO_x_ surface, when Ti was initially evaporated onto the surface, it was not fully oxidized, resulting in a highly hydrophilic surface with a contact angle of 9°. However, as time progressed, the Ti surface underwent oxidation after two days, forming a stable TiO_x_ layer. At this point, the contact angle increased to 74°, indicating a transition from being highly hydrophilic to less hydrophilic. Regarding the PDMS surface, due to the O_2_ plasma treatment and RIE used during fabrication, the surface transformed from being hydrophobic with a contact angle of 99° to being hydrophilic with a contact angle of 12°. However, over time, the contact angle gradually changed from 12° to 45° after two days. It is important to note that even though the contact angle increased, the PDMS surface remained hydrophilic and had no significant changes during the period when cells were monitored as they migrated on the platforms. This indicates that the adhesion of proteins or serum did not cause significant alterations in the surface hydrophilicity of PDMS. Micrographs of the patterned TiO_x_ arrowheads with smooth edges of 10, 20, 35, and 60 μm arm length are shown in Fig. 1(g–j). TiO_x_ exhibits uniform thickness and possesses long-term stability. X-ray photoelectron spectroscopy (XPS) was used to study the chemical compositions of the oxide, and the TiO_x_ was found to have the composition of TiO_1.4_.

The fabrication technology for PDMS grating, as depicted in Supplementary Fig. S2, involved a combination of photolithography and soft lithography techniques. First, patterns were transferred onto a Si wafer using photolithography. Specifically, SPR6112B positive photoresist was spin-coated onto the Si wafer for 1 min at a rotation speed of 3000 rpm. Subsequently, the photoresist-coated wafer was exposed to UV light for 6 s through a photomask. To remove the exposed photoresist, the Si wafer was immediately immersed in a developer solution for 25 s. Following this, the patterned Si wafer was dried and baked on a hotplate at 120 °C for 10 min. The resulting Si wafer with 10 nm deep patterns were formed by RIE. To ensure easy release of the PDMS grating from the Si stamp, the patterned Si stamp was coated with trichloro(1 H,1 H,2 H,2 H-perfluorooctyl)silane (FOTS). Next, PDMS was poured onto the FOTS-treated Si stamp. The PDMS-coated stamp was then baked on a hotplate at 80 °C for 6 h to facilitate curing of the PDMS. As a result, PDMS platforms with the desired patterns were obtained. Finally, the PDMS platforms containing the desired patterns were placed onto a cell culture dish for time-lapse imaging.

The platforms were fabricated using typical semiconductor microfabrication technology. This manufacturing process is well developed and allows for easy mass production of the platforms. Moreover, the resulting platforms exhibit a high level of reproducibility and consistency in their performance. Additionally, it enables the integration of multiple channels in parallel, facilitating high-throughput cell guidance and impedance analysis of numerous cell migration paths. Furthermore, this manufacturing microfabrication technology can be extended to create guidance cues and impedance sensors on complex 3D platforms. This enables real-time monitoring of cell migration within intricate 3D microenvironments. Overall, the microfabrication technology employed in this study offers advantages in terms of simplicity, scalability, consistency, reproducibility, and ability to form 3D biomimetic platforms. These factors contribute to the platform’s suitability for high-throughput directional cell guidance and impedance monitoring of cell migration.

### Cell culture and seeding

MC3T3-E1 osteoblastic cells (ATCC numbers CRL-2594) were cultured in high glucose Dulbecco’s modified eagle medium (DMEM, Invitrogen, USA), supplemented with 10% fetal bovine serum (FBS, Gibco, USA), antibiotic-antimycotic (100 units/ml of penicillin, 100 mg/ml of streptomycin, and 0.25 mg/ml of amphotericin B, Gibco, USA), and 2 mM alanyl-L-glutamine (Gibco, USA). Cells were incubated at 37 ℃ and 5% CO_2_, with the medium changed every 2 days. Prior to cell seeding, the PDMS substrates were washed with 70% alcohol and PBS twice. The MC3T3-E1 cells were seeded at a density of 2 × 10^4^ cells/cm^2^ and allowed to attach on the designed platforms by incubating the culture dish at 37 ℃ in 5% CO_2_ in an incubator for 6 h. The culture medium was then replaced with CO_2_-independent medium (Invitrogen 18045-088, USA) supplemented with 10% FBS, antibiotic-antimycotic, and 2 mM alanyl-L-glutamine (Gibco, USA) for time-lapse imaging.

### Cell migration trajectory, speed, and morphology analysis

The Manual Tracking plugin of Image J software (Version 1.48v, NIH, USA) was used to analyze the migration trajectory, speed, and morphology of MC3T3-E1 in time-lapse images. Images were captured every 5 min for a duration of 16 h using an upright microscope (Eclipse Ni-E, Nikon, Japan) equipped with a 20× objective lens. The cells were maintained in an incubation chamber at 37 ℃ throughout the imaging process. Only cells that remained alive, did not undergo division, and had no interaction with other cells during the 16 h imaging period were included in the analysis. Data were collected from at least three independent experiments. Statistical analysis was performed using one-way analysis of variance (ANOVA) followed by Tukey’s post hoc test to determine significant differences between groups.

### Cell imaging using scanning electron microscopy

After time-lapse imaging, cells were fixed with 4% paraformaldehyde (PFA, Sigma Aldrich, USA) for 15 min. The platform was then washed with 1% and 0.25% PBS for 5 min each, and then rinsed twice in DI water for 10 min each. Subsequently, cells were dehydrated using a series of increasing ethanol concentrations (30%, 50%, 70%, 80%, 95%, and 100%) for 5 min at each concentration. Cells were dried using a critical point dryer. A thin layer of gold was then sputter-coated on the platform using a thin film coater (Q150 coater, Quorum Technologies Ltd., UK), which provided a conductive surface for imaging. High-resolution images of the fixed cells were captured using a field emission scanning electron microscope (SEM, SU5000 FE-SEM, Hitachi, Japan) with a 10 kV electron beam. These images were used to analyze the cell morphology, providing insight into the structure and organization of the cells on the platform.

### Immunofluorescence staining and confocal microscopy

After conducting time-lapse imaging, MC3T3-E1 cells were washed twice with 1% PBS for 5 min each time, and then fixed using 4% PFA for 15 min at 25 ℃. The fixed cells were subsequently washed twice with 1% PBS for 10 min each time, and permeabilized with 0.1% Triton X-100 (Thermo Fisher, USA) for 15 min. After washing the dish twice with 1% PBS for 5 min each time, cells were blocked with 1% bovine serum albumin (Sigma Aldrich, USA) at 25 ℃ for 30 min. The cells were then incubated with primary antibody, mouse anti-vinculin (1:200, Merck, USA) for 12 h, followed by washing with 1% PBS twice. After washing, cells were incubated with the secondary antibody, FITC-conjugated goat anti-mouse IgG (1:500, Merck, USA) for 2 h at 25 ℃. The cells were then washed twice with 1% PBS followed by staining for F-actin using TRITC-conjugated Phalloidin (1:200, Merck, USA) for 2 h. After washing cells twice with 1% PBS, they were stained for the nuclei using DAPI (1:500, Merck, USA) for 30 min. The immunofluorescence images were captured using a confocal microscope (Stellaris 8, Leica, Germany) equipped with a 40× oil objective. The fluorescence signals of the nucleus, vinculin, and F-Actin were imaged using 405, 488, and 532 nm laser, respectively, and marked by blue, green, and red colors. Reflected bright field images were captured using 650 nm laser.

Focal adhesions (FAs) play a crucial role in cellular communication between cells and the extracellular matrix as well as in actin cytoskeleton organization [41]. These adhesive structures are essential for cell adhesion and are critical in cell migration. To evaluate the number, distribution, and area of FAs per cell, ImageJ software was used [42]. The vinculin staining images were first converted to 8-bit files, and then underwent image processing techniques including background removal and FA boundary enhancement, using the built-in Subtract Background function and Contrast Limited Adaptive Histogram Equalization (CLAHE) plugin. The images were further filtered by the Laplacian of Gaussian or Mexican Hat (LOG3D) plugin to identify FAs and eliminate noise, and then converted to binary format with a visually determined threshold to improve the accuracy of FA site detection. The resulting images were then analyzed using the built-in Analyze Particles function to obtain the number and area of FAs. Additionally, the aspect ratio of the cell nucleus was calculated as the ratio between the major and minor axes of the ellipse that best fit the nucleus.

### Microfluidic device with microelectrodes

Microelectrodes consisting of 10 nm Cr layer and 100 nm Au layer were patterned on a glass substrate using the liftoff technology, as shown in Supplementary Fig. S3(a). Micrographs of the microelectrodes with patterned TiO_x_ are presented in Supplementary Fig. S3(b). The microelectrode width and gap between the microelectrodes were both set to 3 μm, and the sensing region measured 480 × 400 μm [2]. External wires were soldered onto the Au pads and sealed using PDMS. The glass substrate with microelectrodes was then glued to a cell culture dish using a thin layer of PDMS.

To create the silicon (Si) stamps for the microfluidic channels, standard photolithography was used with positive photoresist and deep RIE, as described in previous studies [43, 44]. The microfluidic channels were produced using imprint technology. A well-mixed PDMS (base:crosslinker weight ratio = 10:1) was poured onto the Si stamp coated with FOTS and degassed in a vacuum chamber at 10^− 2^ bar for 30 min. The master stamp, along with the uncured PDMS, was then baked at 80 ℃ for 6 h to cure the PDMS. After peeling off from the master stamp, the patterned PDMS device was cut into individual PDMS chips and punched for liquid inlets and outlets using a 0.5 mm biopsy punch.

The surface of the PDMS microfluidic device and the glass substrate containing the microelectrode were treated with an O_2_ plasma at 200 mTorr, 20 sccm, and 50 W RF power for 30 s. Finally, the PDMS microfluidic device was aligned and bonded to the glass substrate containing the microelectrodes and mounted onto a 35 mm confocal dish for optical imaging and impedance measurement. Cells were injected continuously into the microfluidic device. To apply the excitation electric field and measure the electrical responses through the microelectrodes, an impedance analyzer (Reference 600 + Potentiostats, Gamry Instruments, USA) was used.

## Results and discussion

### TiO_x_ in grating shape provided bidirectional guidance for cell migration

**Fig. 2:**
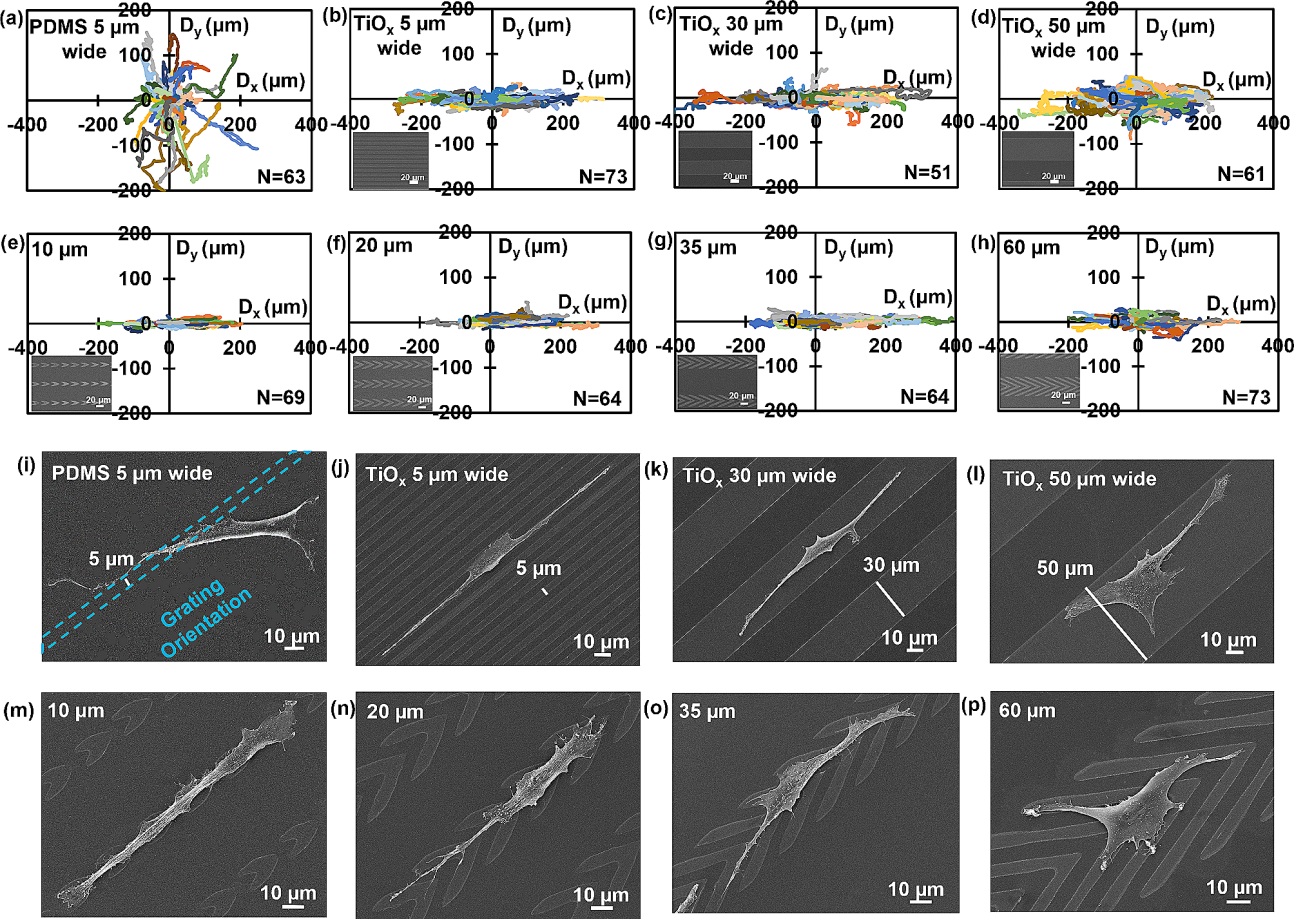
Dependence of cell migration behavior, morphology, and arrowhead shapes with varying arm lengths. Directional migration trajectories of MC3T3-E1 cells on (**a**) PDMS gratings with 10 nm height and 5 μm width; and on 10 nm thick patterned TiO_x_ in grating shape with widths of (**b**) 5, (**c**) 30, and (**d**) 50 μm, and arrowhead shape with arm lengths of (**e**) 10, (**f**) 20, (**g**) 35, and (**h**) 60 μm are shown. Morphology of MC3T3-E1 cells with different shapes on (**i**) PDMS gratings with 10 nm height and 5 μm width; and on patterned TiO_x_ in grating shape with widths of (**j**) 5, (**k**) 30, and (**l**) 50 μm, and arrowhead shape with arm lengths of (**m**) 10, (**n**) 20, (**o**) 35, and (**p**) 60 μm are also presented. The scale bar represents 10 μm

PDMS surfaces patterned with 10 nm thick TiO_x_ in grating shape were fabricated, following the procedure described in Sect. 2.1. Figure 2(a-d) display the migration trajectories of MC3T3-E1 cells on patterned TiO_x_ in grating shape. The shallow PDMS grating with 10 nm height and 5 μm width was fabricated as a control group. Cells on PDMS gratings with 10 nm height and 5 μm width moved randomly, indicating that shallow gratings did not guide cell migration, as shown in Fig. 2(a). This result aligns with previous research, which found that the effectiveness of guiding cell migration with gratings decreased as the height decreased from 4.5 μm to 150 nm [21]. Cells on the gratings with a height of 150 nm and a width of 6 μm showed random movement similar to that on the flat surface. In contrast, cells on patterned thin TiO_x_ in grating shape with widths of 5, 20, and 50 μm exhibited clear directional migration along the grating orientation, as shown in Fig. 2(b-d). These results demonstrate that the difference in surface energy between PDMS and TiO_x_ can constrain cell migration on the surface of patterned TiO_x_ and the polarization direction of MC3T3-E1 cells, which leads to directional cell migration. This finding is consistent with previous studies demonstrating that surface energy can regulate cell responses [13]. 

To visualize cell morphology on different surfaces, SEM was used and representative micrographs were included in Fig. 2(i-l). Compared to cells on PDMS gratings with 10 nm height and 5 μm width, cells on the patterned TiO_x_ in grating shape of the same width exhibited better alignment along the grating orientation. Cells were in contact with both TiO_x_ and PDMS surfaces due to the larger width of MC3T3-E1 cells. However, when the width of the patterned TiO_x_ in grating shape was increased to 30 or 50 μm, cells were constrained to migrate only over the patterned TiO_x_ and not on the PDMS surface. Furthermore, cells on the patterned TiO_x_ in grating shape had a symmetrical shape between the leading and trailing regions, likely responsible for the bidirectional movement of the cells across the grating, as demonstrated in several previous studies [17, 19]. 

Supplementary Fig. S4 shows the migration directionality and speed of the MC3T3-E1 cells on different platforms. Supplementary Fig. S4(a) shows the cell migration speed separately, with V_x_ in the x-direction and V_y_ in the y-direction. All gratings were oriented along the x-direction. The deviation angle θ represents the cell migration directionality on the platforms. At 45°, cells moved with equal speed in both the x- and y-directions, indicating they move randomly without orientation preference. A smaller angle means cells were better guided along the x-direction, which was the grating orientation. Directional migration speed is obtained by calculating the average cell migration speed, V_x_ or V_y_, in either the x- or y-migration direction. PDMS gratings with 10 nm height and 5 μm width did not guide cell migration, while patterned 10 nm thick TiO_x_ in grating shape with 5 μm width induced cell migration along the grating orientation. The directionality of cell migration increased as the width of the grating decreased from 50 to 5 μm. On the other hand, when cells were cultured on patterned TiO_x_ in grating shapes with widths of 5, 30, and 50 μm, their migration speeds were measured to be 0.66, 0.80, and 0.72 μm/min, respectively. These speeds were notably higher than the migration speed observed on PDMS grating, which was 0.38 μm/min. However, cell migration speed was fastest on patterned TiO_x_ in grating shape with 30 μm width, as shown in Supplementary Fig. S4(b). This finding indicates that migration speed and directionality were not significantly correlated.

These findings suggest that the patterned 10 nm thick TiO_x_ surface can significantly influence the behavior of MC3T3-E1 cells by regulating their alignment and morphology. Moreover, cell migration can be controlled by a thin layer of patterned TiO_x_, superior to that of physical contact guidance alone. However, the current geometry of patterned TiO_x_ in grating shape is not effective in controlling cell migration along the desired direction, indicating a need for further improvement. In addition, widening the TiO_x_ pattern beyond 10 μm can provide more effective constrain for cell migration. These results have important implications for the design of future biomaterials and tissue engineering applications.

### Asymmetric arrowhead biased movement of MC3T3-E1 cells

To control cell migration in a desired direction, patterned TiO_x_ in arrowhead shape was developed and analyzed for its effects on unidirectional cell migration. Cell migration trajectories show that cells were limited to move along the patterned TiO_x_ arrowheads in the x-axis direction, similar to cells on the patterned TiO_x_ in grating shape. Notably, cells on arrowheads with 10, 20, and 35 μm arm lengths exhibited migration trajectories along the tips of the arrowheads, as demonstrated in Fig. 2(e-g). However, no significant unidirectional bias was observed for cells on arrowheads with a 60 μm arm length, as shown in Fig. 2(h). Thus, the arrowhead arm length plays a crucial role in regulating unidirectional cell migration.

The cellular response to the patterned thin TiO_x_ in arrowhead shape was further examined using micrographs obtained by SEM, as shown in Fig. 2(m-p). Notably, cells exhibited a preference for spreading and adhering onto the TiO_x_ surface as compared to the PDMS surface. Furthermore, cells on the patterned TiO_x_ in arrowhead shape with 10, 20, and 35 μm arm lengths demonstrated alignment along the direction of arrowhead tips, with the leading regions of these cells protruding toward the tip direction. This is likely due to the increased surface contact area at the arrowhead tips. This phenomenon is consistent with a previous report that showed aligned ratchet-shaped adhesive fibronectin patches inducing biased long-term motion through asymmetric protrusion formation, even in the absence of topographic ratchet patterns [45, 46]. Furthermore, studies have shown that the geometry of the cell adhesive microenvironment plays a crucial role in directing cell surface polarization and internal organization [47, 48]. Therefore, the arrowhead-shaped geometry may align the cytoskeleton and induce cell polarization towards the direction of the arrowhead tip, thus establishing a preferred direction. However, as the arm length of the arrowheads increased to 60 μm, cells tended to protrude along the longer arm, resulting in the reduced probability of cells migrating along the arrowhead tips. Arrowheads with longer arm length had a diminished influence on unidirectional cell migration guidance.

**Fig. 3:**
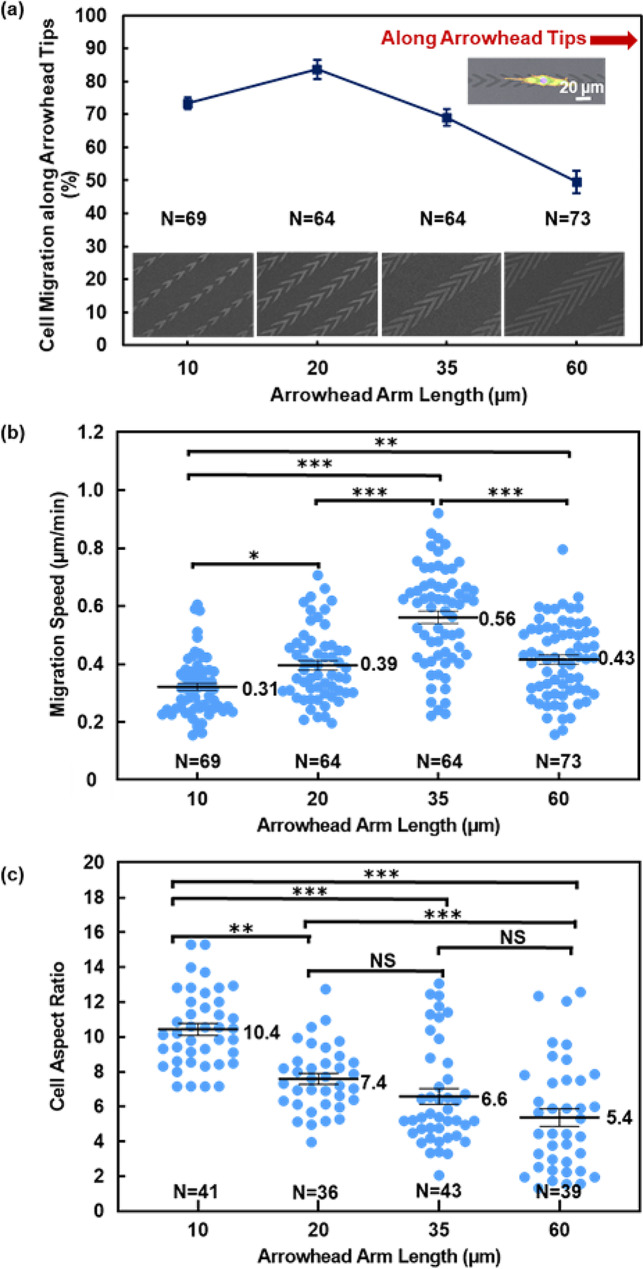
Analysis of directional cell migration behaviors. (**a**) Probability of MC3T3-E1 cells migrating along tips of 10 nm thick TiO_x_ arrowheads with arrowhead arms of 10, 20, 35, and 60 μm in length. The probability was calculated as percentage of cells that migrated along arrowhead tips out of total number of cells that migrated on patterned surface. (**b**) Average migration speed of MC3T3-E1 cells on patterned TiO_x_ arrowheads with arrowhead arms of 10, 20, 35, and 60 μm in length. (**c**) Aspect ratio of MC3T3-E1 cells on different platforms. Aspect ratio was calculated as ratio of cell’s major axis to its minor axis. Numbers in box charts indicate mean values and lines indicate median values. Statistical analysis was performed using one-way ANOVA followed by Tukey’s post hoc test, with **p < 0.01, ***p < 0.001, and NS – not significant

To gain a better understanding of the migration directional preference of MC3T3-E1 cells on patterned TiO_x_ in arrowhead shape with varying arm lengths, the probability of cell migration along the tips of the 10 nm thick TiO_x_ arrowheads was investigated, as shown in Fig. 3(a). The probability of cell migration along the arrowhead tips was calculated by counting the percentage of cells traveling along the arrowhead tip directions among all the cells. When the length of the arrowhead arms was 60 μm, the probability of cell migration along arrowhead tips was 49%, indicating a lack of preference for migration in a single direction. However, for arrowhead arms was ≤ 35 μm, most cells migrated toward the direction of the arrowhead tip. Notably, for arrowhead with 20 μm long arms, 84% of the cells migrated along the arrowhead tips, demonstrating the first-ever unidirectional cell migration control using an ultrathin patterned TiO_x_ layer. Additionally, cells on arrowheads with arm lengths of 10 and 35 μm showed a probability of 73% and 69%, respectively, of migrating in the direction of arrowhead tip. These results suggest that MC3T3-E1 cells can sense the anisotropic geometry of the arrowhead patterns and modify their morphology and migration in response to the TiO_x_ pattern [25]. Hence, these findings offer valuable insights into the interaction between cells and the patterned TiO_x_ layer, which can facilitate the design of biointerfaces with ultrathin patterns for controlling cell migration. This innovation harnesses the combined effects of surface energy and asymmetric topographical cues, offering a pathway to achieve precise control over cell migration direction. The explorations have not only expanded our comprehension of these intricate mechanisms but have also unveiled new opportunities for the precise manipulation and control of cell movement, with potential applications in diverse fields, including biomedicine and tissue engineering.

Surface energy of the platforms is a crucial factor in regulating cell migration. Plasma treatment has conventionally been employed to modify surface energy, but its effectiveness diminishes over time. In contrast, our study utilizes semiconductor manufacturing microfabrication technology to deposit TiO_x_ on PDMS surface, providing a more convenient, reliable, large scale, and precise method for controlling coating thickness and surface energy. Importantly, this approach results in long-term stability. Previous studies have focused on fabricating micropost arrays with stiffness gradients to guide cell migration [9]. For instance, 70% of bovine aortic endothelial cells seeded on a stiffness gradient micropost array exhibited displacement toward regions of increasing stiffness relative to their initial positions. Similarly, by introducing a grating pattern parallel to the direction of the methoxy poly(ethylene glycol) (mPEG) gradient, 67% of vascular smooth muscle cells migrated in a single direction toward the region with lower mPEG density [10]. However, these methods have limitations in terms of coverage, as they could only span over a short distance due to the finite dynamic range of cellular sensing. To overcome this limitation, a microfabricated scaffold with a triangular shape pattern was developed to induce unidirectional cell migration [49]. This resulted in 77% of embryonic mouse fibroblast NIH3T3 cells aligned with the micropattern and migrated in one direction. Additionally, ratchet-shaped micropattern was used to bias cell migration, resulting in 68% of non-cancerous fibroblast Rat2 cells migrating along the ratchet tip direction [50]. In contrast, our study achieved an impressive 84% of cells having unidirectional migration using only an ultrathin layer of patterned TiO_x_ arrowheads, without the need for physical constraints or chemical gradients. The results show the efficacy of our approach in achieving precise unidirectional control over cell migration.

Figure 3(b-c) presents the evaluation of migration speed and cell aspect ratio of MC3T3-E1 cells on various platforms. Migration speed of cells on patterned TiO_x_ arrowheads with 10, 20, 35, and 60 μm arm lengths were measured to be 0.31, 0.39, 0.56, and 0.43 μm/min, respectively, as shown in Fig. 3(b). Interestingly, cells on arrowheads with 35 μm arm length exhibited the highest migration speed of 0.56 μm/min, consistent with the trend observed for cells on patterned TiO_x_ in grating shape. Previous studies have suggested that FAs play a crucial role in cell migration, and the higher cell speed on patterns with acute angles could be attributed to the larger number and area of the FAs per cell [22]. 

In contrast, cells on arrowheads with 10 μm arm length exhibited the slowest migration speed of 0.31 μm/min. During migration, MC3T3-E1 cells showed cyclic behavior characterized by elongation through the protrusions of the leading and trailing regions, detachment of the trailing region from the surface, followed by contraction with cell moving forward [51]. Analysis of cell morphology revealed that shorter arm lengths led to greater cell constraints, resulting in longer cell elongation and migration cycles, ultimately leading to reduced cell migration speed. This observation could explain why cell speed slowed down as the arm length decreased from 35 to 10 μm. However, when the arm length increased to 60 μm, cell migration speed decreased compared to cells on patterned arrowheads with 35 μm arm length. This can be attributed to the cells forming a rounder shape with protrusions in multiple directions for arrowheads with 60 μm arm length, which reduced cell migration speed [52]. It is noted that there is no significant correlation between cell migration directionality and speed.

To gain further insight into the impact of arm length on cell morphology, cell aspect ratio was determined by analyzing the micrographs. Cell aspect ratio was calculated as the ratio between the major and minor axes of cells when they were fitted into an ellipse. As shown in Fig. 3(c), the mean values of the cell aspect ratio were found to be 10.4, 7.4, 6.6, and 5.4 on patterned TiO_x_ arrowheads with 10, 20, 35, and 60 μm arm lengths, respectively. It was observed that the cell aspect ratio decreased as arm length of the arrowheads increased. MC3T3-E1 cells on arrowheads with 10 μm arm length were significantly more elongated than those on arrowheads with longer arm lengths. This implies that with increasing arm length, cell elongation along the arrowhead tip direction decreased, while cells broadened in all directions. These findings emphasize the visibly imperative correlation between cell aspect ratio and alignment with decreasing arm lengths, contributing to a better understanding of cell migration.

Guiding cell migration to the targeted sites is a key strategy in tissue repair, particularly in cases of severe bone defects where regeneration remains a significant challenge. To address this issue, the development of regenerative scaffolds that direct tissue growth has emerged as a potential solution. In this study, TiO_x_ arrowheads were patterned onto a PDMS surface. The highly biocompatible TiO_x_ surface led to cell migration predominantly on the hydrophilic TiO_x_ surface, while the asymmetrical arrowhead tips provided larger surface contact areas to control migration direction. Consequently, this platform design facilitated cell protrusion and migration along the arrowhead tips, allowing precise control over unidirectional cell migration. The ultrathin TiO_x_ arrowhead pattern was effective in guiding oriented tissue growth. By assembling these TiO_x_ patterns in a strategic manner, complex 3D bionic scaffolds can be created, effectively guiding cell migration from the distal end to the proximal end of the scaffold and promoting directional tissue growth. This study serves as a foundation for the construction of intricate 3D biomimetic scaffolds for guided growth of tissues.

### Distribution of focal adhesions determined cell migration direction

To better understand the mechanisms behind unidirectional cell migration on patterned TiO_x_ arrowheads, staining was performed to examine the MC3T3-E1 cell morphology, FA, and cytoskeleton. Specifically, staining was carried out for the nucleus, vinculin, and actin fibers. As depicted in Supplementary Fig. S5(a-c), a majority of cells on patterned TiO_x_ arrowheads with arm lengths of 10, 20, and 35 μm exhibited a polarized morphology, stretching uniaxially along the direction of arrowhead tips. This was achieved by cells actively sensing the ultrathin TiO_x_ arrowhead pattern with the asymmetrical geometry. These results highlight the remarkable ability of cells to migrate on patterned thin TiO_x_ surface, as opposed to the PDMS surface. Conversely, cells on arrowheads with 60 μm arm length spread out more broadly on the TiO_x_ surface, as shown in Supplementary Fig. S5(d).

FAs preferentially formed on TiO_x_ surface rather than PDMS surface when cells moved on patterned arrowheads, which may contribute to why cells were guided to migrate on arrowhead pattern. Moreover, cells on the patterned TiO_x_ arrowheads with 20, 35, and 60 μm arm lengths formed more FAs around the cell bodies than on arrowheads with 10 μm arm length. This may contribute to the faster migration speed for cells on the patterned TiO_x_ arrowheads with 20, 35, and 60 μm arm lengths. In addition, when the length of the arrowhead arm was ≤ 35 μm, actin fibers assembled in the direction of the arrowhead tips and cells protruded lamellipodia in the same direction. Filopodia and lamellipodia are actin-rich protrusions that allow cells to probe the surrounding surface while searching for locations to make focal contacts [52]. Thus, the patterned arrowheads restricted the alignment of cells on the patterned TiO_x_ surface, and the asymmetrical geometry of the arrowheads caused the cells to protrude along the arrowhead tip direction, resulting in the unidirectional cell migration.

**Fig. 4:**
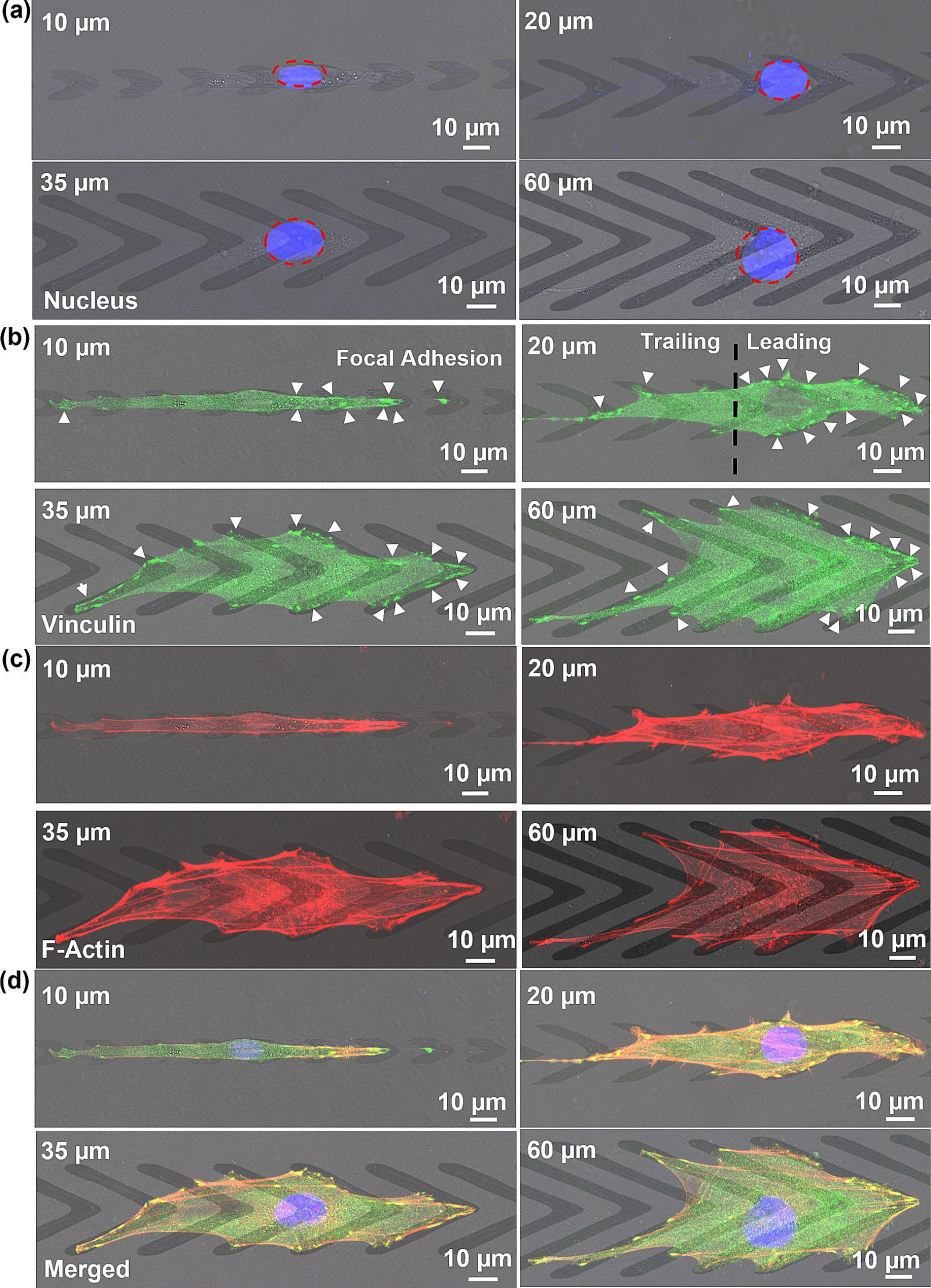
Immunofluorescence micrographs of MC3T3-E1 cells cultured on patterned TiO_x_ in arrowhead shapes with various arm lengths. All cells were stained to observe (**a**) nucleus, (**b**) focal adhesions (FAs), and (**c**) actin fibers under fluorescent imaging. (**d**) Cell nuclei were merged with the fluorescent images of vinculin and filament actin (Blue: nucleus, Green: vinculin, and Red: filament actin). Cells were equally divided into two regions, with leading region in direction of arrowhead tip and trailing region in opposite direction. White arrows point to FAs of MC3T3-E1 cells

**Fig. 5:**
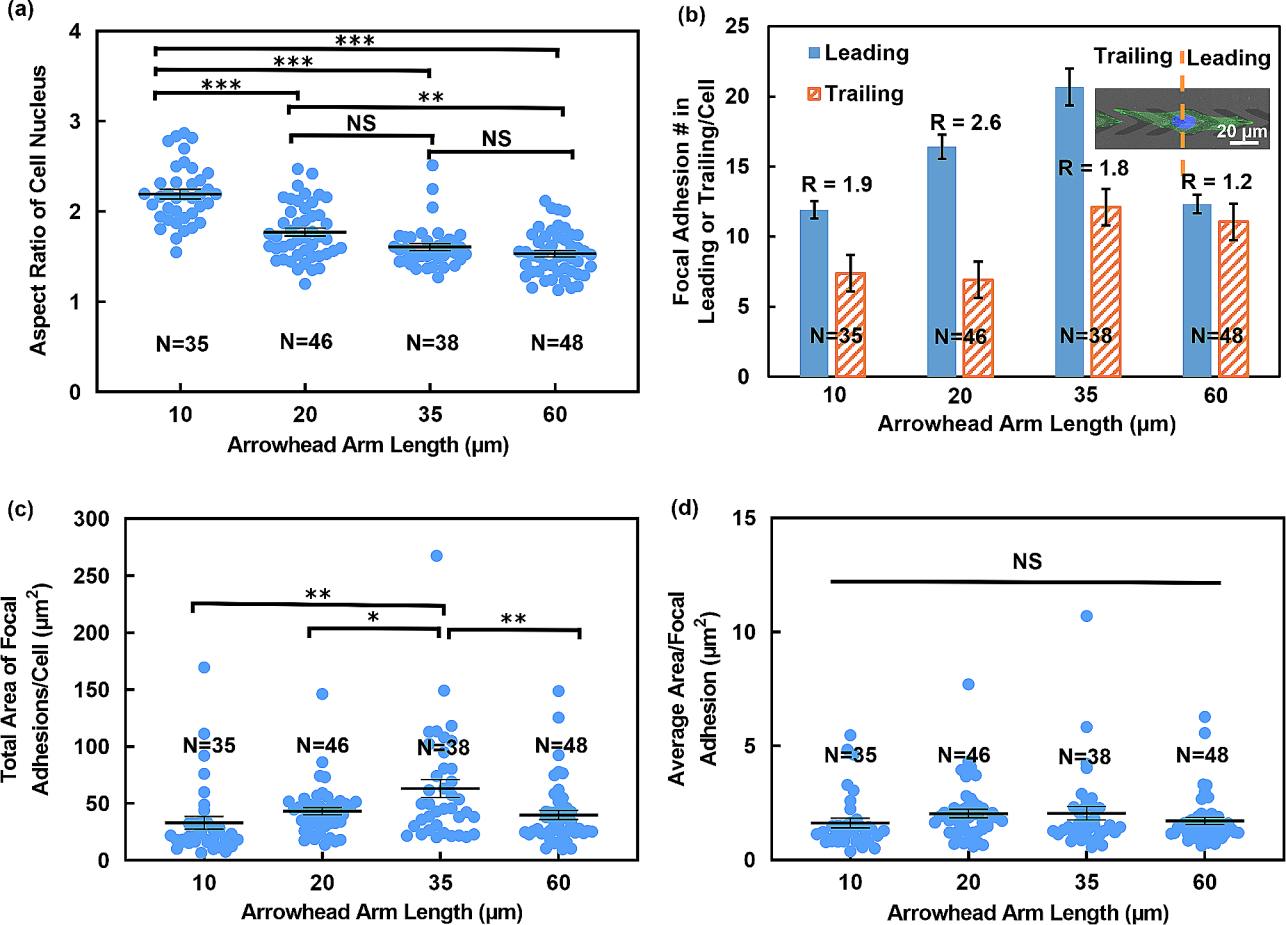
Analysis of FAs and cell nuclei in cells cultured on patterned TiO_x_ in arrowhead shapes with various arm lengths from immunofluorescence micrographs. (**a**) Aspect ratio of cell nucleus. (**b**) Distribution of FAs on leading and trailing regions of cells. R represents ratio of number of FA between leading and trailing regions. (**c**) Total area of FAs per cell. (**d**) Average area per FA. Statistical analysis was performed using one-way ANOVA followed by Tukey’s post hoc test, with **p < 0.01, ***p < 0.001, and NS – not significant

Micrographs in Fig. 4 shows the cell nucleus, vinculin, and F-actin on patterned TiO_x_ arrowheads. As the length of the arrowhead arm decreased, cells exhibited a more confined alignment along the patterned TiO_x_ orientation, resulting in a narrower nucleus morphology, as shown in Fig. 4(a). On the patterned arrowheads with 10, 20, 35, and 60 μm arm length, the aspect ratio of cell nucleus was 2.2, 1.8, 1.6, and 1.6, respectively, as shown in Fig. 5(a). This suggests a correlation between nucleus shape, cell aspect ratio, and arm length. As previously reported, a dome-like actin structure referred to as an “actin cap” was found to cover the top of the nucleus [53]. This actin structure plays a role in the deformation of the nucleus shape. The deformation and orientation of nuclei in response to cell shape changes were primarily governed by lateral compression forces. When a cell elongated, the stress fibers on either side of the nucleus exerted lateral compression force, regulating the shape of the nucleus. Meanwhile, the apical actin filaments exerted vertical compression force and constrained the height of the nucleus.

Additionally, FAs only formed on the TiO_x_ surface, and no FAs formed on the PDMS surface of the gaps between adjacent arrowheads, as shown in Fig. 4(b). Before cells were seeded in the platforms, there was no coating of cell adhesion molecules. The hydrophilicity of the PDMS and TiO_x_ surfaces were maintained as shown in Supplementary Fig. S1. The self-secreted adhesion molecules and adhesion molecules from FBS in the culture medium may preferentially bind to the TiO_x_ surface, resulting in more FA formation. Subsequently, the cells migrated along the direction of the patterned TiO_x_ arrowhead tips with higher FA concentrations near the tips, thereby achieving unidirectional cell movement. It is important to note that if the entire platform is coated with adhesion molecules prior to cell seeding, the surface energy difference between the PDMS and TiO_x_ surfaces would be compromised. This would diminish the ability of the TiO_x_ arrowheads to guide unidirectional cell migration. By intentionally maintaining the surface energy contrast between the PDMS and TiO_x_ surfaces, the designed platforms maximize the unidirectional guiding effect of the patterned TiO_x_ arrowheads.

Cells were divided evenly along the arrowhead tip direction, with the leading region corresponding to the region along the arrowhead tips and the trailing region being the other half of the cell, as indicated in Fig. 4(b). When the arrowhead arm lengths were ≤ 35 μm, most FAs were concentrated around the leading region of cells, indicating an anisotropic distribution of FAs induced by the asymmetrical geometry of the arrowheads. In contrast, when the arrowhead arm length was 60 μm, the majority of FAs were located around the cell periphery, with nearly equal numbers between the leading and trailing regions. Figure 5(b) shows that MC3T3-E1 cells on arrowheads with 10 and 20 μm arm lengths typically exhibited 12 and 16 FAs/cell at the leading region, respectively, and an equal number of 7 FAs/cell at the trailing region. For cells on arrowheads with 35 μm arm length, there were 21 FAs/cell at the leading region and 12 FAs/cell at the trailing region. Cells on arrowheads with 60 μm arm length had 12 FAs/cell at the leading region and 11 FAs/cell at the trailing region, and the number of FAs/cell was nearly identical.

Furthermore, the ratio (R) of FA numbers between the leading and trailing regions was calculated and shown in Fig. 5(b). For cells on arrowheads with 10, 20, and 35 μm arm lengths, the FA ratio between the leading and trailing regions was 1.9, 2.6, and 1.8, respectively, which were significantly higher than the ratio of 1.2 observed for cells on arrowheads with 60 μm arm length. These results indicate that patterned thin TiO_x_ arrowheads with arm lengths of 10, 20, and 35 μm exert control over cell migration direction by inducing asymmetrical distribution of FAs. This asymmetrical distribution of FAs promotes cell adhesion and protrusion along the direction of the arrowhead tips, thereby initiating unidirectional cell migration. Increased formation of actin-rich protrusions along the arrowhead tip direction generated an imbalanced traction force, thereby facilitating unidirectional cell migration [54]. In contrast, FAs on arrowheads with 60 μm arm length were more evenly distributed, resulting in no pronounced directional preference for cell movement.

The total number of FAs per cell for cells on patterned arrowheads with 10, 20, 35, and 60 μm arm length were 19, 23, 33, and 23, respectively, as calculated from Fig. 5(b). The total FAs area per cell also showed a similar trend, with values of 33.1, 43.3, 63.3, and 39.9 μm [2], respectively, for cells on patterned arrowheads with 10, 20, 35, and 60 μm arm length, as shown in Fig. 5(c). Notably, there was no significant difference in the average area per FA, as shown in Fig. 5(d), which may be attributed to FAs forming on the TiO_x_ surface of the same surface energy. Previous studies have shown that different surface energies can modulate the size and morphology of FAs, but cells on surfaces with the same surface energy exhibited similar FA formation and distribution [14, 15, 55, 56]. These findings suggest that the total number and total area of FAs per cell significantly influence migration speed, especially when there is no significant difference in the average area per FA. Overall, these results demonstrate the crucial role of FAs in regulating cell migration behaviors.

FAs are known to connect with stress fibers. Compared to cells on patterned TiO_x_ arrowheads with 60 μm arm length, cells on the arrowheads with 10, 20, and 35 μm arm lengths exhibited aligned actin fibers in the arrowhead tip direction and preferentially formed actin fibers at the periphery of cells, as depicted in Fig. 4(c). Actin fibers were observed to form between the FAs around the cells, resulting in the compression of the nuclei and increases in cell aspect ratio [57]. The merged images of the nucleus, vinculin, and F-actin were shown in Fig. 4(d). Results from our previous study [17] show that FAs were mostly aligned with the patterned structures but showed random distribution on plane surface. While the shape of FAs was similar, but the size of FAs was larger on patterned surface compared to plane surface. Cell migration on a plane surface is random and the leading regions can protrude in all directions. In our platforms with patterned arrowheads, without the presence of external forces like fluid flow, asymmetrical ultrathin TiO_x_ arrowhead pattern was utilized to guide the formation of FAs. This results in higher concentrations of FAs forming around the tips of the arrowheads, leading to cell polarization and protrusion of the leading regions in the directions of the arrowhead tips. As a result, the probability of cell migration reversal is reduced. This pattern design promotes cell migration along the arrowhead tips, enabling the precise control of unidirectional cell migration. Overall, these results indicate that cell migration speed and morphology are regulated by the formation of FAs and stress fibers [58]. 

### Monitor individual cell migration using electrical impedance measurements

In our aforementioned study, the analysis of cell migration behaviors relies on expensive and complex microscopes, as well as manual tracking and calculations, which can be both costly and time-consuming. Therefore, there is a pressing need for a simple and automated monitoring and analysis method. Impedance monitoring offers several advantages, including non-destructive, real-time data acquisition and analysis, miniaturization, and automation, making it highly valuable for studying cell migration. However, a challenge arises when integrating PDMS biomimetic platforms with topographic cues with electrodes for impedance measurement, as the thick PDMS guiding patterns can compromise sensitivity. To address this issue, a method utilizing ultrathin TiO_x_ patterns directly deposited on electrodes was developed, enabling impedance monitoring of cells under guided migration. This achievement marks the first instance of leveraging this combination of impedance monitoring on platforms with thin layers of guiding patterns to study the dynamics of cell migration. The interdigital microelectrodes were fabricated using technologies described in Supplementary Fig. S3. The microelectrodes were integrated with patterned TiO_x_ in arrowhead shape.

**Fig. 6:**
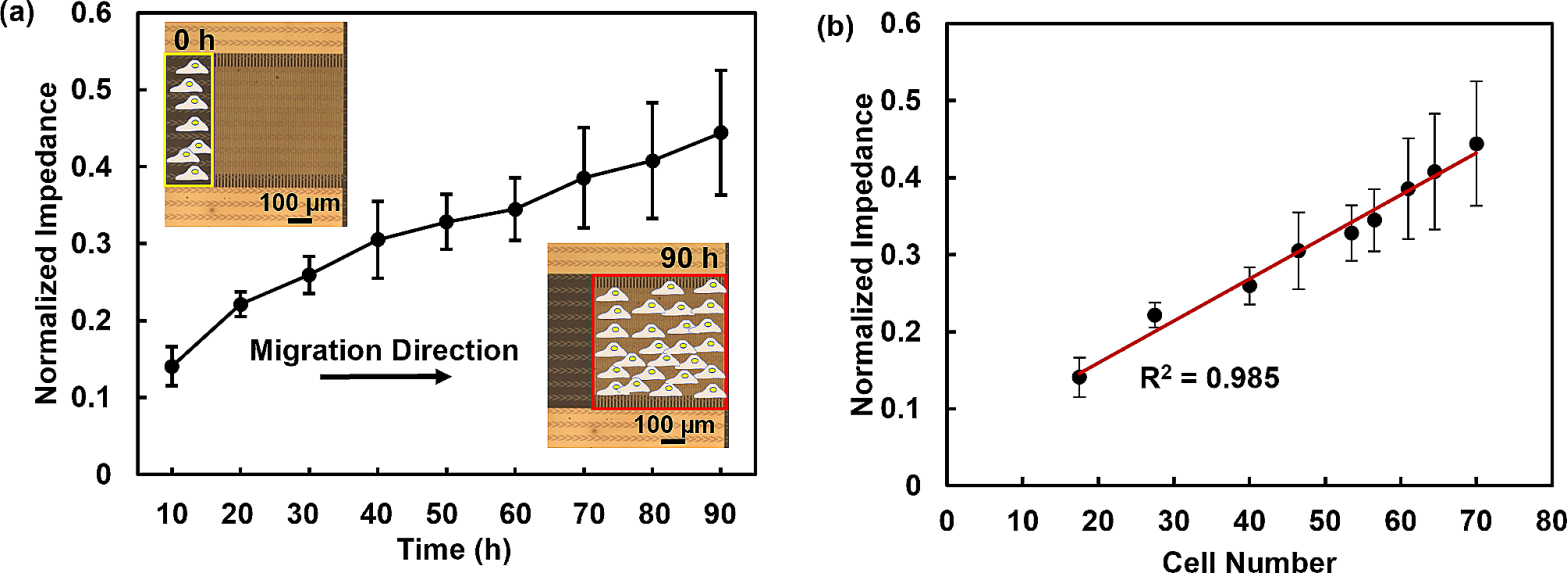
Impedance monitoring of cell migration using interdigital electrode integrated with patterned TiO_x_. (**a**) Normalized impedance versus time for real-time impedance monitoring of MC3T3-E1 cell migration over 90 h. (**b**) Correlation between normalized impedance and number of cells after seeding for 10 h. Cells were initially seeded on edge of sensor region

To enable initial seeding of cells at the edge of the sensing region, a PDMS block was used to cover the sensing area at 0 h. After 6 h, the PDMS block was removed, and cells began to migrate along the patterned TiO_x_ arrowheads. The impedance was continuously monitored using an impedance analyzer at 100 mV and 1 MHz. The results of real-time impedance monitoring of MC3T3-E1 cell migration over 90 h is presented in Fig. 6(a), showing that the normalized impedance of MC3T3-E1 cells increased with time. The relationship between the impedance response and cell number is shown in Fig. 6(b). As the cell number increased and cells gradually covered the surface of the interdigital electrodes, the normalized impedance value increased. This is primarily due to the insulating cell membrane, which leads to an increased interface impedance when cells migrate on the electrode surface [59]. However, changes in the impedance due to the presence of cells cannot be observed when the number of cells is small. Thus, these interdigital microelectrodes are not sensitive enough for single-cell analysis.

**Fig. 7:**
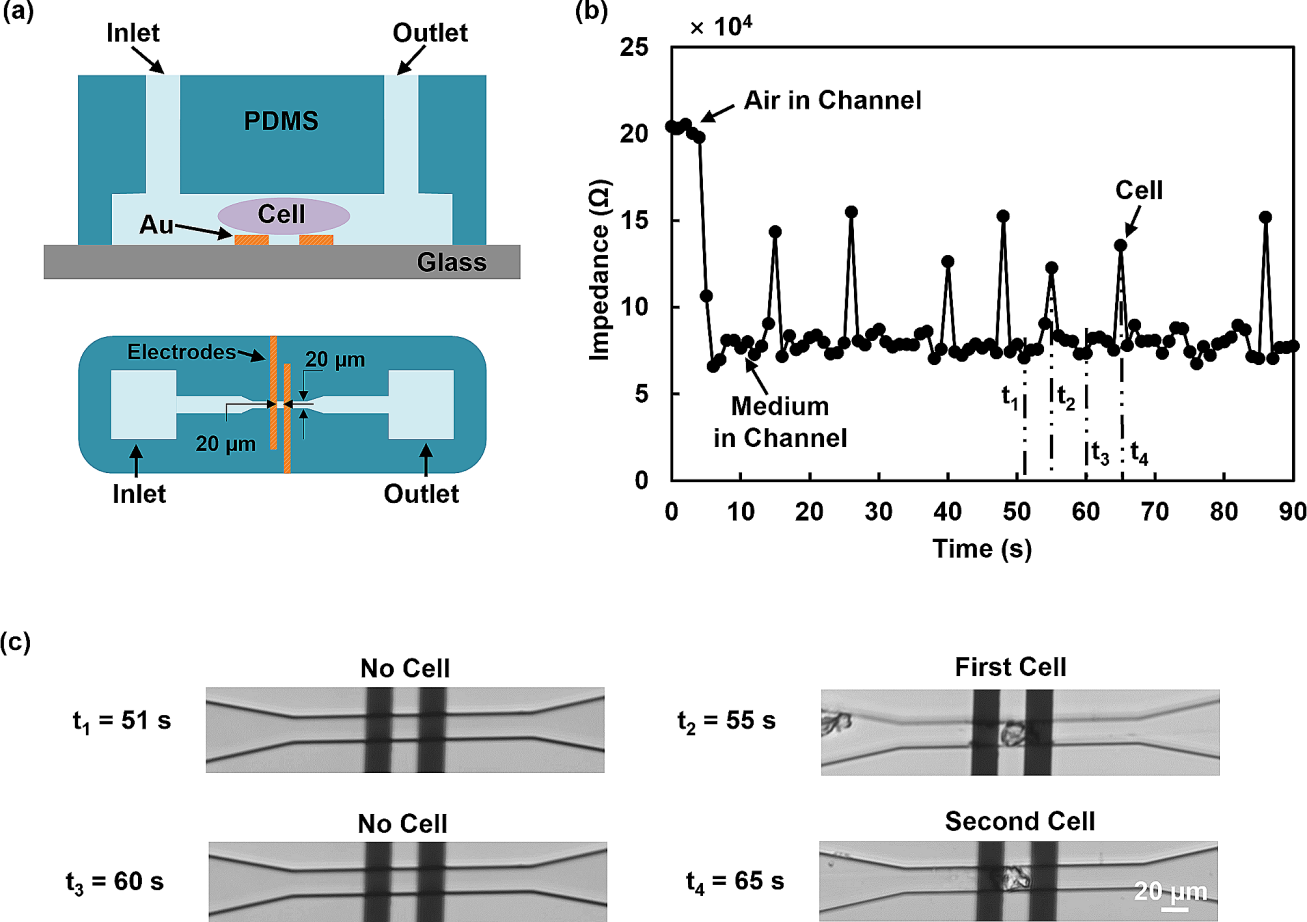
Cell monitoring using impedance measurement at 1 MHz and 100 mV. (**a**) Schematic of microfluidic system for electrical characterization of single cells using impedance spectroscopy and narrow channel. (**b**) Electrical impedance signal changed as single cells passed through electrodes. (**c**) Time-lapse micrographs of single MC3T3-E1 cells passing through electrodes

The microfluidic channel combined with the microelectrodes allowed for the characterization of individual biological cells by measuring their electrical properties, as shown in Fig. 7(a). The channel was designed to fit an individual MC3T3-E1 cell and was made of PDMS, with dimensions of 20 μm in width, 15 μm in height, and 20 μm between electrodes. The channel was aligned and bonded to the two coplanar electrodes on the glass substrate. A pump was used to drive the cells through the channel at 10 nL/min. Impedance was measured over time using an impedance analyzer, as shown in Fig. 7(b). Sequential microscopic images of the cell positions at different time points were recorded, as shown in Fig. 7(c). For each cell passing through the electrodes, an impedance peak appeared when the cell was midway between the electrodes. In continuous flow, each impedance peak corresponded to a passing cell. When no cell touched the electrode at t_1_ and t_3_, the impedance value was lower as the cell medium was conductive. When the first and second cell passed through the narrow channel at t_2_ and t_4_, the impedance was higher compared to the case without a cell [60]. This impedance measurement allowed for the characterization of individual cells and their electrical properties, which is important for a range of applications in biomedical studies and clinical diagnostics. The observed peak values were not uniform, which can be attributed to the intrinsic heterogeneity of the cells. Different cell morphologies or sizes with varying contact areas with the microelectrode could lead to different impedance values [61]. Overall, this microfluidic device enables reliable single-cell analysis based on the electrical properties of cells and has great potential for real-time cell monitoring. By integrating impedance measurements in the platforms with a combination of microelectrodes and guiding patterns, and analyzing cell migration characteristics such as speed, position, and directionality, it is possible to achieve automated monitoring and analysis of cell migration without relying on optical imaging and manual tracking. In conclusion, electrical impedance measurements offer an automated technique for monitoring cellular responses to extracellular stimuli. This approach can speed up the cell migration data analysis and provide valuable insights for cell-based assays and contribute to advancements in biomedical and pharmacological research.

TiO_x_ is commonly utilized as a coating for bone scaffolds to enhance cell adhesion and promote cell differentiation. However, the conventional approach of completely covering the bone scaffold with a TiO_x_ coating does not provide the directed migration of cells in the areas with the desired tissue growth. Our developed fabrication technology allows patterning of ultrathin TiO_x_ layers, enabling directed cell migration while avoiding polymer reactions or cracking. Furthermore, the designed ultrathin TiO_x_ patterns can be integrated to form a complex 3D scaffold to mimic tissues in vivo. Impedance measurements offer advantages over conventional optical measurements as it is difficult to capture cell migration in a complex 3D scaffold using an optical microscope. Additionally, the electrical measurement facilitates the miniaturization and automation of the cell migration monitoring system. Therefore, impedance measurements allow monitoring of cell migration within complex 3D platforms, which is not easily achievable using optical imaging.

## Conclusions

PDMS platforms with patterned 10 nm thick TiO_x_ layers were developed to investigate their effects on controlling cell migration. The effects of pattern geometry and physical dimensions on cell migration behaviors were studied. The results showed that cells on PDMS gratings with a height of 10 nm and a width of 5 μm displayed random movement, similar to cells on a flat surface. However, cells on patterned 10 nm thick TiO_x_ in grating shape were constrained to align with the grating orientation and achieved bidirectional migration due to the difference in surface energy between PDMS and TiO_x_. To control cell migration in a single direction, patterned TiO_x_ in arrowhead shape was fabricated. The 10 nm thick arrowhead shape patterns induced strong directional guidance to the cells when the length of the arrowhead arms was ≤ 35 μm. The larger contact area of arrowhead tips led to an imbalance in cell polarization, resulting in unidirectional cell migration along the tip direction. These results indicated that ultrathin TiO_x_ pattern with the asymmetrical geometric shape could mediate cell migration direction.

Moreover, the relationship between cell migration behaviors, arrowhead arm length, and FAs was analyzed. For cells on arrowheads with 10, 20, and 35 μm arm lengths, the ratio of FA number between leading and trailing regions was 1.9, 2.6, and 1.8, respectively, which were much higher than that of the cells on arrowheads of 60 μm arm length with a ratio of 1.2. These findings indicated that patterned thin TiO_x_ arrowheads with arm lengths of 10, 20, and 30 μm could control cell migration by triggering FA formation with asymmetrical distributions. The asymmetrical FA distribution promoted cell adhesion and protrusion along the arrowhead tips, which resulted in unidirectional cell migration. Additionally, cell migration speed was related to the total number and area of FAs. Furthermore, the longer the cell elongated, the narrower the nucleus, which also affected the cell migration speed. These findings highlight the potential of proper biointerface designs to guide cell migration unidirectionally, which could have significant applications in tissue repair and regeneration.

Our research demonstrates that the patterning of TiO_x_ arrowheads onto PDMS platforms promotes osteoblast cell protrusion and migration along the arrowhead tips, resulting in precise control over unidirectional osteoblast cell migration. To further expand on this topic, future studies include investigation of how different cell types would respond to these platforms. It is widely known that diverse cell types exhibit varying responses when exposed to the same surfaces. Cell adhesion to biomaterial surfaces affects cell migration, proliferation, and differentiation, all of which play crucial roles in tissue regeneration [62]. Cell adhesion depends on the complex interplay between receptors and ligands, which occur on the surfaces of cell membranes and biomaterials. While different cell types would initiate different cellular signaling pathways when they come into contact with the platforms, the asymmetrical arrowhead pattern design is expected to induce more FA near the arrowhead tips, which could result in unidirectional cell migration.

On the other hand, interdigital microelectrodes integrated with patterned TiO_x_ in arrowhead shape were employed to monitor cell migration. It was observed that the normalized impedance increased as the cell number increased when cells progressively covered the surface of the microelectrodes. However, these interdigital microelectrodes were not sensitive enough for single-cell analysis. To overcome this limitation, a microfluidic channel combined with a pair of microelectrodes was developed. This setup allowed for the monitoring of the electrical properties of individual biological cells. Notably, when a cell passed through the electrodes, an impedance peak occurred. In continuous flow of cells, each peak in the impedance corresponded to a passing cell. This microfluidic device with cell migration guiding arrowhead pattern enables real-time cell detection based on the electrical properties of cells, opening up possibilities for cell sorting and screening.

### Electronic supplementary material

Below is the link to the electronic supplementary material.

Supplementary Material 1

## Data Availability

No datasets were generated or analysed during the current study.
