# Supplementary material for: Biointerfaces with ultrathin patterns for directional control of cell migration
Source: J Nanobiotechnology. 2024 Apr 8;22:158. doi: 10.1186/s12951-024-02418-3 (PMC11000378; doi:10.1186/s12951-024-02418-3)
Supplement: Supplementary file 1 — Supplementary Material 1 [file 12951_2024_2418_MOESM1_ESM.pdf]

Electronic Supplementary Information (ESI)

Biointerfaces with Ultrathin Patterns for Directional Control of Cell Migration

Yijun Cheng[1,2] and Stella W. Pang[1,2]*

[1]Department of Electrical Engineering, City University of Hong Kong, Kowloon, Hong Kong, China

[2]Centre for Biosystems, Neuroscience, and Nanotechnology, City University of Hong Kong, Kowloon, Hong Kong, China

*Corresponding Author: pang@cityu.edu.hk

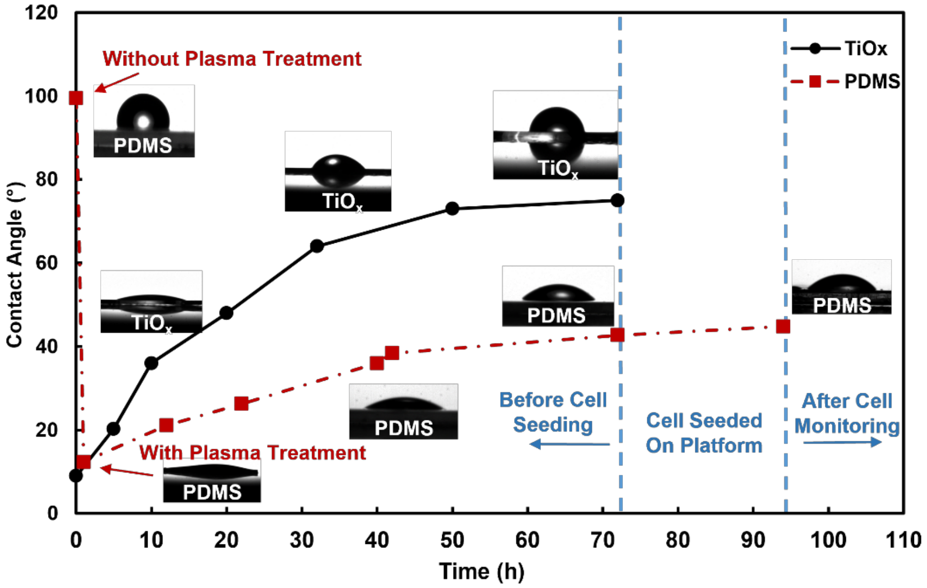

Supplementary Figure S1. Measurements of changes in contact angles for different surfaces over time.

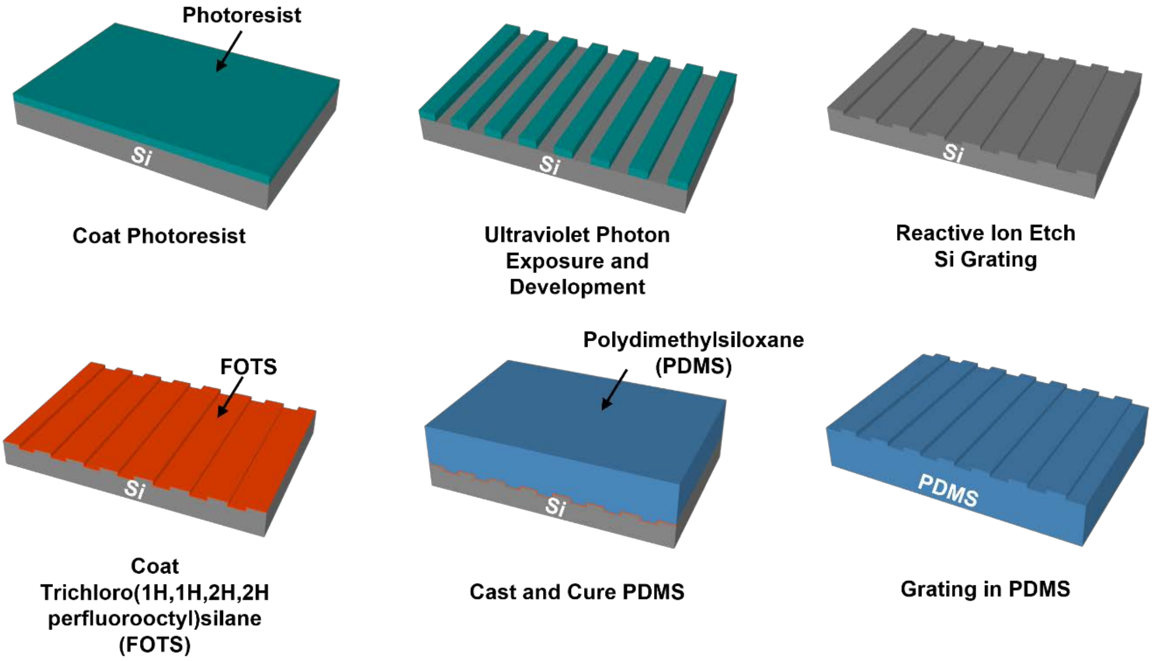

Supplementary Figure S2. Schematics of fabrication technology for PDMS grating.

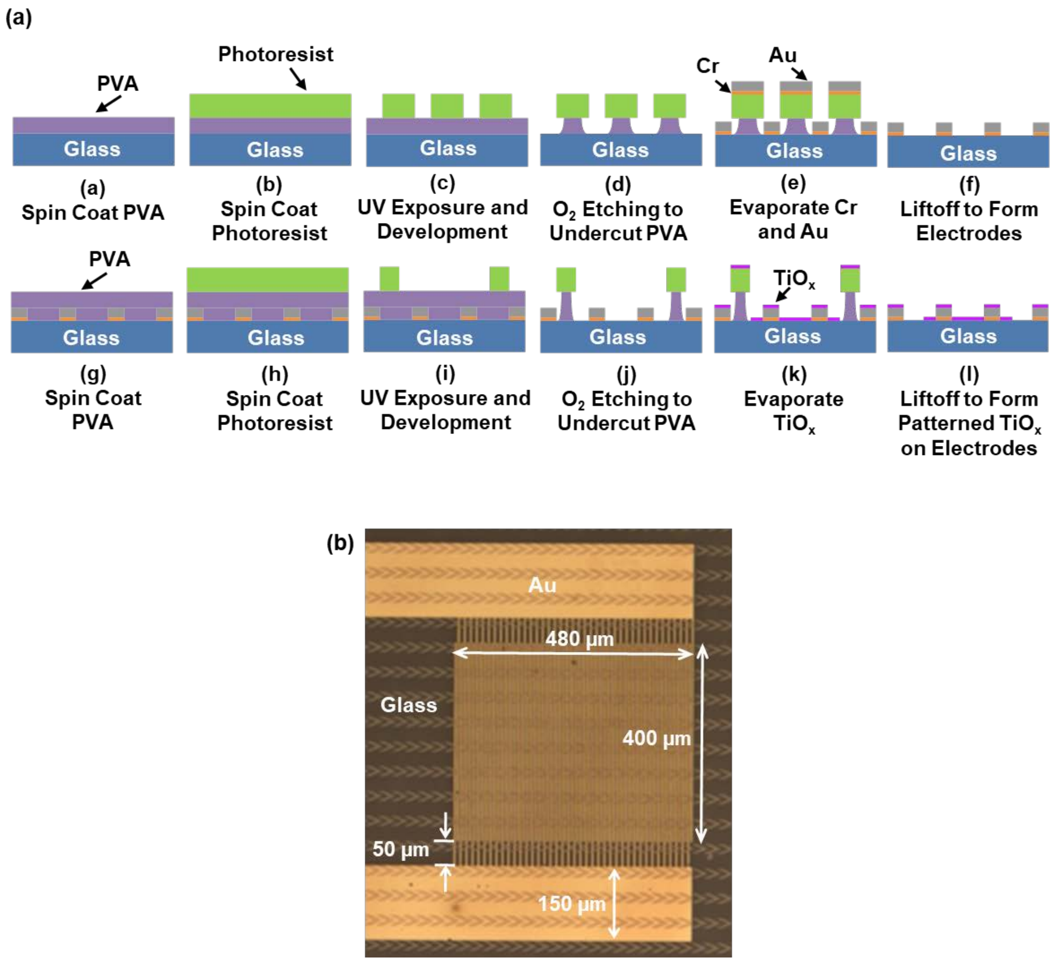

Supplementary Figure S3. (a) Schematics of fabrication technology and (b) optical micrograph for interdigital microelectrode integrated with patterned TiO_x.

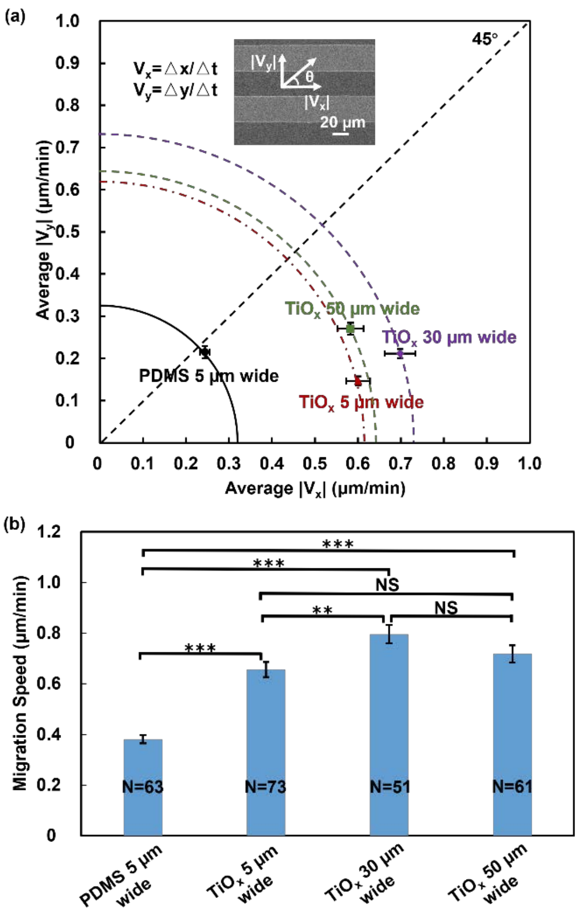

Supplementary Figure S4. (a) Migration directionality of MC3T3-E1 cells on PDMS gratings with 10 nm height and 5 µm width; and on patterned TiO_x in grating shape with 5, 30, and 50 µm widths. All gratings were oriented along x-direction as shown in inset. (b) MC3T3-E1 cell migration speed on PDMS with 10 nm height and 5 µm width; and on patterned TiO_x in grating shape with 5, 30, and 50 µm widths. Statistical analysis was performed using one-way ANOVA followed by Tukey's post hoc test, with $^{**}p < 0.01$, $^{***}p < 0.001$, and NS – not significant.

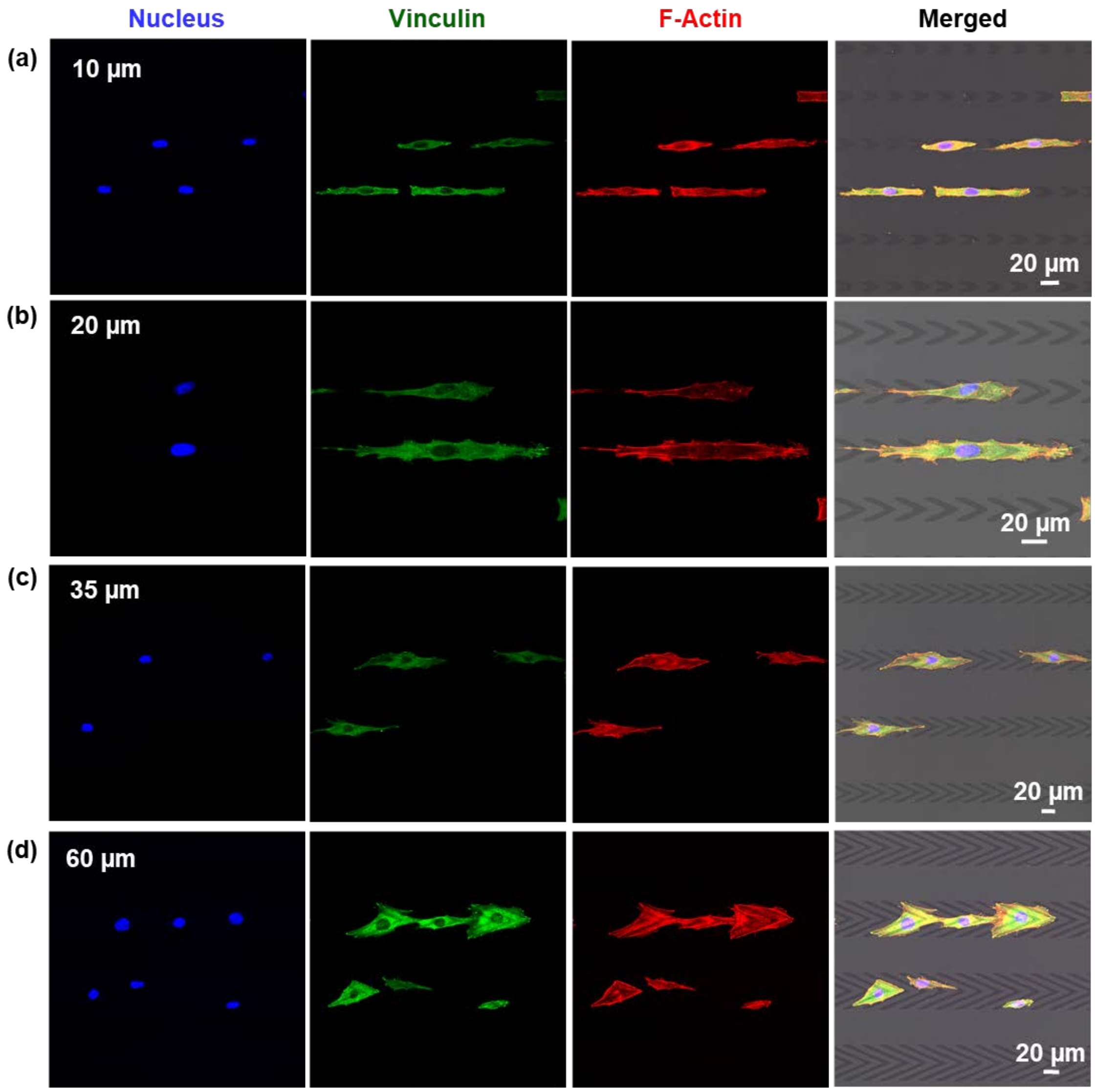

Supplementary Figure S5. Immunofluorescence images of MC3T3-E1 cells cultured on patterned TiO_x in arrowhead shape with arm lengths of (a) 10, (b) 20, (c) 35, and (d) 60 μm. (Blue: nucleus, Green: vinculin, and Red: filament actin).
